# Identification of Novel and Conserved microRNAs in *Homalodisca vitripennis*, the Glassy-Winged Sharpshooter by Expression Profiling

**DOI:** 10.1371/journal.pone.0139771

**Published:** 2015-10-06

**Authors:** Raja Sekhar Nandety, Almas Sharif, Shizuo G. Kamita, Asokan Ramasamy, Bryce W. Falk

**Affiliations:** 1 Department of Plant Pathology, University of California, Davis, California, United States of America; 2 Department of Entomology & Nematology, University of California, Davis, California, United States of America; 3 Division of Biotechnology, Indian Institute of Horticultural Research, Bangalore, India; Queen's University Belfast, UNITED KINGDOM

## Abstract

The glassy-winged sharpshooter (GWSS) *Homalodisca vitripennis* (Hemiptera: Cicadellidae), is a xylem-feeding leafhopper and an important vector of the bacterium *Xylella fastidiosa;* the causal agent of Pierce’s disease of grapevines. MicroRNAs are a class of small RNAs that play an important role in the functional development of various organisms including insects. In *H*. *vitripennis*, we identified microRNAs using high-throughput deep sequencing of adults followed by computational and manual annotation. A total of 14 novel microRNAs that are not found in the miRBase were identified from adult *H*. *vitripennis*. Conserved microRNAs were also found in our datasets. By comparison to our previously determined transcriptome sequence of *H*. *vitripennis*, we identified the potential targets of the microRNAs in the transcriptome. This microRNA profile information not only provides a more nuanced understanding of the biological and physiological mechanisms that govern gene expression in *H*. *vitripennis*, but may also lead to the identification of novel mechanisms for biorationally designed management strategies through the use of microRNAs.

## Introduction

MicroRNAs (miRNAs) are a class of non-protein coding small RNAs that are known to play important roles in post-transcriptional level gene regulation and expression (reviewed in [1,2]. MicroRNAs are typically 19–25 nt in length and are the most abundant class of endogenous small RNAs in both plants and animals [3]. MicroRNAs can also originate from either non-coding or coding regions of transcripts. The primary transcripts of microRNAs are predominantly cleaved and processed within the cell nucleus by the nuclease Drosha to generate precursor microRNAs (pre-miRNAs) with a characteristic hairpin like secondary structure. The pre-miRNAs are then transported into the cytoplasm by Exportin–5, where the terminal loop of the pre-miRNA is removed by the ribonuclease Dicer–1 (Dcr–1) producing a miRNA-miRNA star duplex with 2 nucleotide overhangs at both ends. The miRNA-miRNA star duplex becomes incorporated into the RNA Induced Silencing Complex (RISC) in which Argonaute–1 (Ago–1) protein is the main component. The miRNA guide strand and the Argonaute protein form the core of the RISC complex that helps in the recognition of the miRNA target. The miRNA star strand (passenger strand) is then degraded and the other miRNA strand (guide strand) guides the RISC complex to the target mRNA [4]. In some cases, both the miRNA and miRNA star strands are retained as functional microRNAs [5]. Recent studies indicate that there is a 5’ bias for the first nucleotide that can influence the type of Argonaute protein into which the microRNA can load in the RISC complex [6]. The recognition of the microRNA target is facilitated through the microRNA seed sequence that stretches from the second through seventh nucleotides [6]. One of the striking features of microRNAs is the arrangement of their precursors in a cluster format, that can often result in the production of more than one microRNA from the same primary transcript [7]. MicroRNAs regulate gene expression by either direct pairing through their *cis*-regulatory roles or as *trans*-acting small interfering RNAs. For example, a recent microRNA functional study shows that the silkworm ecdysone receptor (EcR) is regulated by microRNA–281 in an isoform specific manner [8]. Similarly, in the hemipteran insect *Nilaparvata lugens* ecdysone-induced chitin biosynthesis is regulated by the conserved microRNAs miR-8-5p and miR-2a-3p [9]. MicroRNAs also play key roles in insect host-pathogen crosstalk and immunity [10].

Recent advances in sequencing technologies and the development of sophisticated computational algorithms such as miRDeep [11], miRDeep* [12], MIReNA [13] and miRDeep-P [14] have led to the identification of novel microRNAs from plants, animals and insects. Such computational predictions are possible due to the conservation of microRNA sequences and the characteristic hairpin structures of their precursors. The microRNA functions established thus far show that they regulate gene expression by either direct pairing through their *cis*-regulatory roles or as *trans*-acting small interfering RNAs. The complementarity of miRNA and their target mRNA sequences can be used to predict microRNA targets from the available sources of genomic data, such as transcriptomic data in *H*. *vitripennis* [15]. With advances in bioinformatics and with growing interest in microRNA research, a central repository of microRNAs (both conserved and novel) is maintained at miRBase [16,17]. MicroRNA sequencing in insects has resulted in a wealth of information on both conserved and novel microRNAs from insects in several orders including the silkworm, *Bombyx mori* [18]; brown planthopper, *N*. *lugens* [19]; honeybee, *Apis mellifera* [20]; mosquitoes, *Aedes albopictus* and *Culex quinquefasciatus* [21]; German cockroach, *Blattella germanica* [22]; citrus red mite [23]; stable fly, *Stomoxys calcitrans* [24]; cotton bollworm, *Helicoverpa armigera*, tobacco cutworm, *Spodoptera litura* [25]; sexually dimorphic insect *Eupolyphaga sinensis* [26]; whitefly, *Bemisia tabaci* [27]; gall midge, *Mayetiola destructor* [28]; cotton-melon aphid, *Aphis gossypii* [29]; and tobacco hornworm, *Manduca sexta* [30].

The glassy-winged sharpshooter, *H*. *vitripennis*, is a plant xylem-feeding leafhopper that is an economically important pest of a wide range of plants including *Citrus* spp., grapes (*Vitis vinifera*), and almonds (*Prunus dulcis*) [31]. This insect also serves as a robust vector of the bacterium *Xylella fastidiosa*, the causal agent of Pierce's disease of grapevines and citrus variegated chlorosis disease [32]. *H*. *vitripennis* is used as a model for the study of host-insect-plant pathogen interactions due to their feeding nature and the ability to transmit plant pathogens. We have recently determined the transcriptome sequence of *H*. *vitripennis* [15]. Through microRNA sequencing, we sought here to gain a preliminary understanding of miRNAs that are associated with biological processes in *H*. *vitripennis* such as cell proliferation, cell fate specification, cell differentiation, apoptosis, metabolism, molting, metamorphosis, and transposon regulation; and to identify possible targets for RNAi-based insect control. MicroRNA analysis of *H*. *vitripennis* can also provide insights into potential roles of microRNAs in insect biology and pathogen-host interactions; that can generally aid us in understanding the molecular mechanisms of miRNA regulation.

The objectives of this study were to use a combination of deep sequencing of small RNAs of *H*. *vitripennis* and computational analysis to systematically predict microRNA species in the *H*. *vitripennis* and to identify their possible targets. We performed Illumina-based sequencing of small RNAs isolated from adult *H*. *vitripennis* and developed better understanding of the complexity of microRNA regulation in *H*. *vitripennis*. Our analyses identified a number of new and novel microRNAs and their precursor RNAs. Our results provide a global view of the microRNAome of adult *H*. *vitripennis* and pave the way for its further analysis and exploitation.

## Materials and Methods

### Maintenance of *H*. *vitripennis* and total RNA extraction

A colony of *H*. *vitripennis* was maintained at the University of California-Davis Contained Research Facility (CRF) in cages containing a mixture of host plants as previously described [31]. Total RNAs were extracted using TRIzol reagent (Invitrogen, Carlsbad, CA, USA) from eight day-old adults (males and females) or 2^nd^—3^rd^ instar individual nymphs

### Library preparation and sequence data generation

Total RNA (2 μg per library) was used as the template to construct single-end indexed Illumina small RNA libraries. Samples were processed according to Illumina's TruSeq Small RNA sample preparation guide. Briefly, the small RNAs were ligated to Illumina's small RNA 3’ and 5’ adaptors. Subsequently cDNA was synthesized by reverse transcription and amplified by PCR (11 cycles) purified by two size selection gels. Sequencing of the small RNA library was performed by the Illumina Genome Analyzer. The small RNA raw sequence data in the form of a FASTQ file has been uploaded to the SRA database at NCBI (accession number: SAMN03853644; Accession ID: SRS985993) (http://www.ncbi.nlm.nih.gov/biosample/?term=SAMN03853644). The reference numbers for the SRA experiments are SRX1090328 and SRR2095935 (http://www.ncbi.nlm.nih.gov/sra/SRR2095935/). The Bio Project accession number allotted to the sequence information is PRJNA289427 (Accession: SRP060579).

### Data extraction and analysis

All of the Illumina sequencing data were initially converted to FASTA format from FASTQ. Small RNA sequences were extracted from raw reads by removal of the adaptor sequences. Based on the length of the mature miRNA and adapter length, small RNA sequences of 22 nt in length were filtered out, checked for redundancy and were queried against the ribosomal and transfer RNA databases. The sequences were then queried against the *H*. *vitripennis* transcriptome [15] and the sequences that mapped to the transcriptome were retained. The sequences were also aligned against miRBase [16,17,33–36] to identify the conserved (two mismatches allowed) and novel microRNAs. Mature miRNA sequences were used as query against *H*. *vitripennis* transcriptome to locate corresponding precursors that had at least 18 matched base pairs, one central loop and folding energy lower than 18 kCal/mol. The frequency of the miRNAs and miRNA*s were calculated based on the read numbers in the library. All bioinformatics analyses were performed using custom written perl scripts and through the help of the scripts made available through the miRDeep2 software [11]. Further annotation of the microRNA sequences was performed through manual annotation. We were able to identify precursors for the newly identified microRNAs from the transcriptome information of *H*. *vitripennis* [15]. This is important because we did not have to use a proxy reference as suggested in the recent literature on the correct usage of microRNA discovery pipelines in non-model organisms [37].

### MicroRNA target predictions

Target prediction for animal miRNAs is complex because of imperfect complementarity between miRNAs and their mRNA targets. The target candidates for miRNAs of *H*. *vitripennis* were chosen based on our mRNA dataset (~52,700 sequences) that were generated through transcriptome sequencing [15]. The miRNA target candidates were analyzed with target prediction software MiRanda version 3.3a [38]. The three criteria that were allowed for the screening of miRNA target alignments were: no mismatch at the seed region (positions 2 to 7 from the 5’ end of predicted miRNA); not more than 1 G:U paring at the seed region; and not more than 1 gap in the miRNA:mRNA duplex. Except for the energy and score parameters, the remaining parameters in the MiRanda version 3.3a software were set to default settings. The total score cutoff value was set for ≥145 and energy threshold value of ≤ −10 kcal/mol was set as second parameter.

### Quantitative real-time PCR

Total RNA (isolated as described before) from adult and nymphal (2^nd^—3^rd^ instars) *H*. *vitripennis* was used to study the expression pattern of the conserved and novel microRNAs. The miRNA expression was measured using a two-step process. In the first step, a stem-loop (RT) primer (designed based on previous reports [39]) was hybridized to the miRNA and reverse transcribed in a pulsed RT reaction [40]. In the second step, the RT reaction product was PCR amplified using a miRNA specific forward primer and a universal reverse primer (S1 Table) in real time with SYBR green chemistry using a Bio-Rad CFX Real-Time PCR detection system [40]. Quantification of the relative changes in miRNA expression was performed using the method previously described [40]. In this relative quantification method, the sample reference was chosen as miR10a and the endogenous control was chosen as ubiquitin [41]. The data for relative quantities were converted to fold differences by logarithmic transformation to express the data as a normal distribution. The data presented are the averages of three measurements.

### MicroRNA northern blots

Some of the conserved and novel microRNAs were validated using microRNA northern blots through the use of 5’ end labeled probes. Total RNA was isolated from the individual adult *H*. *vitripennis* using Trizol reagent and small RNAs were precipitated using 5 M NaCl and 50% PEG8000 [42]. Briefly, three micro grams of adult *H*. *vitripennis* small RNAs were separated on denaturing 8 M urea–15% polyacrylamide gels and transferred to a nylon membrane (Amersham Hybond™-NX) as described previously [43]. The membrane was then UV cross-linked (Stratagene). Synthetic antisense DNA oligomers were 5’-end labeled using [γ^32^P]-ATP (Perkin Elmer) and T4 Polynucleotide kinase (New England Biolabs) and used as probes for hybridization. The probes were purified on a Sephadex G–25 column (mini quick spin oligo columns, Roche Diagnostics). Prehybridization was carried out for 5 hours using ULTRAhyb-oligo hybridization buffer (Ambion, AM8663). The hybridization was performed using a probe specific activity of 2 x 10^6^ cpm per ml of hybridization buffer. The hybridization was carried out for 16 hours at 42°C in a hybridization oven. Following hybridization, the membrane was washed twice by shaking in low stringency wash solution (2X SSC, 0.1% (w/v) SDS) at 42°C for 20 min. The blots were removed, drained, wrapped in Saran Wrap and exposed to X-ray film at −80°C for 6–8 days. MicroRNA Marker (New England Biolabs, Ipswich, MA) was used on the same gel to estimate RNA sizes. Wild type potato (*Solanum tuberosum* cv. Desiree) small RNA samples on which the insects fed were used as negative controls.

## Results

### Overview of the dataset

Sequencing of the adult *H*. *vitripennis* small RNA library generated 22 million reads. After small RNA read processing, the remaining sequence reads (43% of the total reads) were mapped to an artificial build of the *H*. *vitripennis* transcriptome [15]. Available scripts from the miRDeep2 software package were used to identify novel microRNAs [11]. The miRDeep2 analysis identified microRNA sequences along with their abundance. We were also able to investigate the secondary structure of each potential precursor microRNA as well as the coordinates of those precursor microRNAs in the transcriptome of *H*. *vitripennis*. In the filtering step, potential precursors that were grossly inconsistent with miRNA biogenesis were discarded. Based on the algorithmic scores assigned from the scripts, each potential microRNA precursor was verified for the combined compatibility of energetic stability, position and frequency of reads with Dicer processing.

### Conserved microRNAs in *H*. *vitripennis*

**Fig 1:**
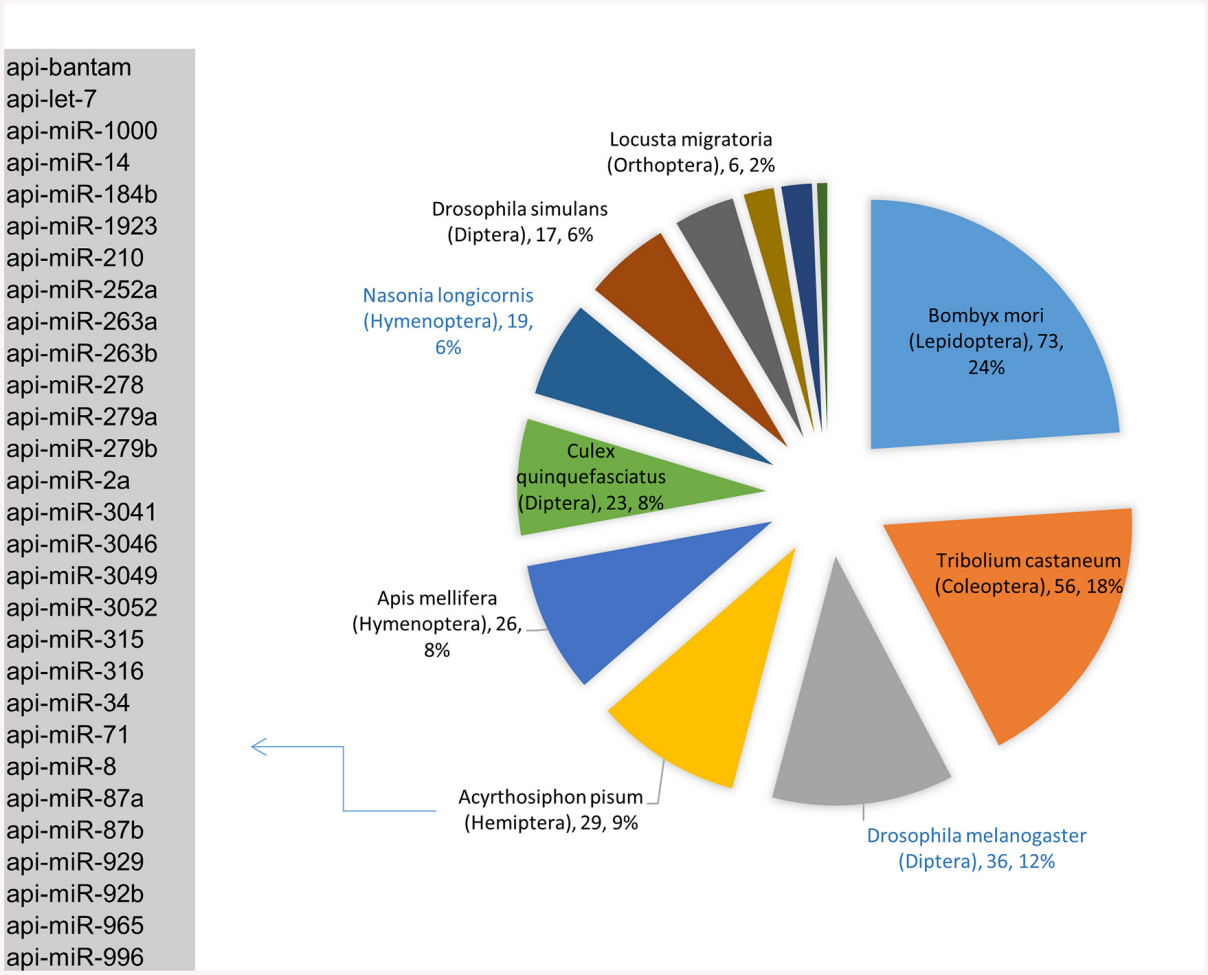
Conservation of microRNA sequences from *H*. *vitripennis* and other insects. The pie diagram shows the number of hits (and percentage of hits) that the conserved microRNAs from *H*. *vitripennis* showed with microRNA sequences from other insects. MicroRNAs from the hemipteran *Acrythosiphon pisum* that were found in common with those of *H*. *vitripennis* are listed in the inset.

In order to identify conserved microRNA sequences, the potential microRNAs from the adult *H*. *vitripennis* were compared to the available miRBase microRNA datasets [36]. Many of microRNAs from adult *H*. *vitripennis* shared homology with those of other insects (Fig 1). The highest conservation in microRNAs was observed with microRNAs from *Bombyx mori* (73), *Tribolium castaneum* (56), *Drosophila melanogaster* (36), *Acrythosiphon pisum* (29), and *Apis mellifera* (26). Six conserved microRNAs were observed in common with the parasitic wasp *Nasonia vitripennis*. The conserved microRNAs observed between *H*. *vitripennis* and the hemipteran *Acrythosiphon pisum* (29) are listed in the inset in Fig 1. In total, 345 microRNAs from *H*. *vitripennis* showed conserved homology with microRNAs of other insects in the miRBase microRNA datasets (Fig 1). The microRNA conservation homology was also observed with *Culex quinquesfasciatus* (23), *Nasonia longicornis* (19), *Drosophila simulans* (17), *Drosophila pseudoobscura* (14), *Aedes aegypti* (12), *Locusta migratoria* (6), and *Anopheles gambiae* (2). The conserved microRNAs observed between *H*. *vitripennis* and *Acrythosiphon pisum* (29) are listed in Fig 1.

### Novel microRNAs in *H*. *vitripennis*

Novel species-specific microRNAs can be identified and annotated on the basis of 1) criteria defined for the identification of novel microRNAs (i.e., the presence of a miRNA-star strand, low-energy, and fold back precursor structure [30,44]), and 2) the lack of resemblance to known microRNAs in the available miRBase database. We used scripts from the miRDeep2 software package [11] to identify novel microRNAs in *H*. *vitripennis*. Because *A*. *pisum* is the closest hemipteran species (based on the available sequence information) to *H*. *vitripennis*, microRNAs from *A*. *pisum* were used as reference sequences. Additional microRNA sequences from *B*. *mori*, *T*. *castaneum*, and *N*. *vitripennis* were used for more accurate guidance. The precursor sequences and structures were identified from the available transcriptome information of *H*. *vitripennis*. Manual analysis of the algorithmic predictions helped us to identify and annotate 14 novel microRNAs (Table 1). All of these sequences have low-energy, fold-back precursor structures. The consensus star strand and the consensus precursor sequences for the microRNAs were identified (Table 2). Interestingly, a majority (11 out of 14) of the novel microRNAs that were identified in *H*. *vitripennis* showed a 5’ terminal uridine bias. This 5’ terminal uridine bias for microRNAs was previously reported to allow for specific Argonaute 1 (AGO1) recognition and loading in the RISC complex. Two of the novel microRNAs, Hvi- miR2062 and Hvi-miR6225, had 5’ terminal guanosine and one Hvi-miR24657 had a 5’ terminal adenosine. Recent studies on miRNA loading show an increase in loading of microRNAs with a 5’ terminal adenosine bias to AGO2 and those with a cytidine bias into AGO5 [6].

**Table 1:** Name and Sequence of the Novel MicroRNAs Identified from *H*. *vitripennis*.

Name	Sequence
Hvi-miR29035	ugcaacaguucuggcuuggcaa
Hvi-miR13059	uuuguucugaauggcacgucgg
Hvi-miR28196	uaguuuacaucaucgacugugc
Hvi-miR9237	uguuugaacaccucggccuuua
Hvi-miR19117	uccaguaguaagccucaaacca
Hvi-miR24402	uuauaguuccuugcgcuacguu
Hvi-miR66	ugauaagucagacauuugaaag
Hvi-miR41359	uagaucuaguucccuucugcug
Hvi-miR29828	ucugcuugcuccguaccuacuu
Hvi-miR2062	gaagaaguacuuggugccguca
Hvi-miR20768	uaugugauucuuguacucggcu
Hvi-miR91	uuuuuguuggaaaccgacaacc
Hvi-miR24657	agaaagacuauugcagagcugc
Hvi-miR6225	ggauaucauagagggacuugaa

**Table 2:** Precursor MicroRNA Sequences of the Novel MicroRNAs Identified from *H*. *vitripennis*.

miRNA	Precursor Sequence
Hvi-miR29035	uuuuacuccauauaacauuguucaaagauucauugggguuuugggauuuaccucugagacauuuuugcaacaguucuggcuuggcaagau
Hvi-miR13059	uuuguucugaauggcacgucggagacugggcaggguugcgcaguaggcuucugaguuaaucgucauuccucgcg
Hvi-miR28196	guuacggggaaucggaauaaaagacugaauuaguuuacaucaucgacugugcucg
Hvi-miR9237	uguuugaacaccucggccuuuaaguaaccccacagaaaaaagucacaugggguuaaaucgggugaucgcgcuggccaca
Hvi-miR19117	uccaguaguaagccucaaaccaaauguuguuguagauuacaacaaguauaugugugggguagacaagcaggacc
Hvi-miR24402	ucuggugcuugugguuggugauaauucuacagcauuuauuauugcuuguucauuauuauaguuccuugcgcuacguu
Hvi-miR66	ugauaagucagacauuugaaagaugcgucgccgguacgaggaccgugcgaucagcugaaaguuauucagagucaccaagaag
Hvi-miR41359	gcagcagaaggaaacuaauacaagaaacaaaaacuuagaucuaguucccuucugcug
Hvi-miR29828	uucuuguagguacauuggagcagcucuuuguaaaggcacucugcuugcuccguaccuacuucga
Hvi-miR2062	gaagaaguacuuggugccgucagacuugacugucggucaguuuuacuucuug
Hvi-miR20768	uaugugauucuuguacucggcuuguauugccucucuuucugacucaaauuaccuuuaaguuuagcuugguaggaaucgcauguauc
Hvi-miR91	uuuggcugucguguuuggaauuaacaaguucaggcguuaccuagaacauaaagaauuuuuguuggaaaccgacaaccagg
Hvi-miR24657	agaaagacuauugcagagcugcagagacagaggcucggcccucauucucagcugcgacugcaaugcucaccacacgu
Hvi-miR6225	guucaagccucuguaugauauaaauguauguauguugacaaaaggauaucauagagggacuugaacuu

### Survey of the potential microRNA encoding genes in *H*. *vitripennis*

A total of 372 mature miRNAs from *A*. *pisum*, *B*. *mori*, *T*. *castaneum*, and *N*. *vitripennis* were used as reference sequences to identify potential microRNA encoding genes in *H*. *vitripennis*. The conserved and newly identified novel microRNA sequences were used to search the transcriptome assembly of *H*. *vitripennis* using scripts from bowtie [45] and mirdeep2 [11] functional algorithms, and through UNIX-based scripts. Under these criteria, a total of 14 transcripts that potentially encoded the newly identified microRNAs were identified from the *H*. *vitripennis* transcriptome (Table 3). The sequence from each locus was used to determine the existence of a stem-loop pre-miRNA structure using mfold [46]. The microRNA target loci annotations from the transcriptome data were used to identify the relevance of the potential origins of the microRNAs in the transcriptome. Based on this analysis, seven (Hvi-miR13059, Hvi-miR9237, Hvi-miR19117, Hvi-miR24402, Hvi-miR41359, Hvi-miR91 and Hvi-miR24657) of the 14 identified microRNAs have origins that correspond to transposable elements (Table 3). Of these seven, Hvi-miR24402 and Hvi-miR9237 originated from loci that belonged to the TC3 type of transposable elements (Table 3). One of the microRNAs that did not originate from a transposable element sequence, Hvi-miR2062, originated from a gamma amino butyric acid (GABA) receptor gene locus [47]. The coordinates of the originating loci of the precursor microRNAs in the transcriptome of *H*. *vitripennis* are presented in Table 3. Energy and fold-back structures of these precursor microRNA sequences, containing the mature strand and the consensus star strand sequences, are shown in Table 4.

**Table 3:** Target Loci of the Novel MicroRNAs from *H*. *vitripennis*.

miRNA	Identified Origin	Coordinates
Hvi-miR29035	Uncharacterized protein with oxidoreductase activity	395–485
Hvi-miR13059	Transposable element	817–891
Hvi-miR28196	Uncharacterized protein	159–214
Hvi-miR9237	TC3 type transposable element	739–818
Hvi-miR19117	Transposable element	125–199
Hvi-miR24402	TC3 type transposable element	877–957
Hvi-miR66	Uncharacterized	188–274
Hvi-miR41359	Transposable element	195–255
Hvi-miR29828	Ion transport protein involved in response to abiotic stress	169–233
Hvi-miR2062	Gamma amino butyric acid receptor	147–199
Hvi-miR20768	Uncharacterized	75–161
Hvi-miR91	Retro transposable element	195–275
Hvi-miR24657	Transposable element	30–107
Hvi-miR6225	Uncharacterized transmembrane protein	737–805

**Table 4:** Predicted Structure of Precursor MicroRNA Sequences of from *H*. *vitripennis*.

miRNA	Precursor Structure^1^
Hvi-miR29035	…….(((..((..(((((((((((((((…(((((……….)))))…)).))))))).))))))..))…)))……
Hvi-miR13059	…((…(((((((((.((((((((((.((……)).))))….))))))))…..)))))))…)).
Hvi-miR28196	..(((((….((((……((((((…))))))……)))))))))….
Hvi-miR9237	…………..((((……((((((((..((……))…))))))))…..((((….))))))))…
Hvi-miR19117	(((.((.((..((((((…((.(((((((((((…)))))))).))).))..))))))..))..)).)))..
Hvi-miR24402	..((((((….((…((((((((…((((((……..))).)))..))))))))….))..))))))….
Hvi-miR66	……….(((.((((((.((((((((((((((……)))).)))))..))…..))).)))))))))………
Hvi-miR41359	.((((((((((.(((((((..(((………)))..)).))))).))))))))))
Hvi-miR29828	…..((((((((…((((((((….(((….)))….)).))))))))))))))…..
Hvi-miR2062	.((((((((……((((.(((……))).))))……)))))))).
Hvi-miR20768	(((((((((((((…..(((…….)))……………………((((…..)))).)))))))))))))….
Hvi-miR91	.((((.(((((.((((….((((((((((((((…..))).))))………..)))))))))))))))).)))).
Hvi-miR24657	…..((.((((((((.((.((.((((..((((……))))..)))).)).))..)))))))).))………
Hvi-miR6225	(((((((.((((.((((((((…….(((……)))…..)))))))).)))).)))))))..

^1^The structures of the precursors are presented in Dot-Bracket Notation (DBN). In the DBN method, dotted positions are unpaired, whereas matching parenthesized positions represent base-pairing nucleotides.

### Abundance of the novel microRNAs

We identified 14 novel microRNAs that varied in their abundance in the library, based on the cut off value of 10. Among these microRNAs, Hvi-miR29828 showed higher abundance (1,860 copies) in comparison to the other novel microRNAs (Table 5). There were three other novel microRNAs; (Hvi-miR29035 (326 copies), Hvi-miR13059 (234 copies) and Hvi-miR91 (145 copies)) each showed more than 100 reads (Table 5). Interestingly, all of the mature microRNAs that were expressed above the threshold level showed a strong 5’ terminal uridine bias, suggesting a strong loading into the AGO1 protein of the RISC complex (Table 1). Eleven of the 14 novel microRNA reads were comprised of mature strand reads and not the star strand reads (Table 5). In the case of Hvi-miR13059, Hvi-miR28196, and Hvi-miR29828, both the mature and star strand reads were found at close to a 1:1 or 2:1 ratio. Comparatively, the novel microRNAs with a bias for a different nucleotide at the terminal 5’ position were identified with either very minimal or no star strand reads.

**Table 5:** Abundance of Novel MicroRNAs from *H*. *vitripennis*.

miRNA	Total Reads	Mature Reads	Star Reads
Hvi-miR29035	326	324	2
Hvi-miR13059	234	112	122
Hvi-miR28196	69	48	21
Hvi-miR9237	23	22	1
Hvi-miR19117	19	18	1
Hvi-miR24402	22	21	1
Hvi-miR66	12	10	2
Hvi-miR41359	13	13	0
Hvi-miR29828	1860	1,007	853
Hvi-miR2062	95	94	1
Hvi-miR20768	16	15	1
Hvi-miR91	145	143	2
Hvi-miR24657	12	10	2
Hvi-miR6225	24	24	0

### Expression of microRNAs in *H*. *vitripennis*

**Fig 2:**
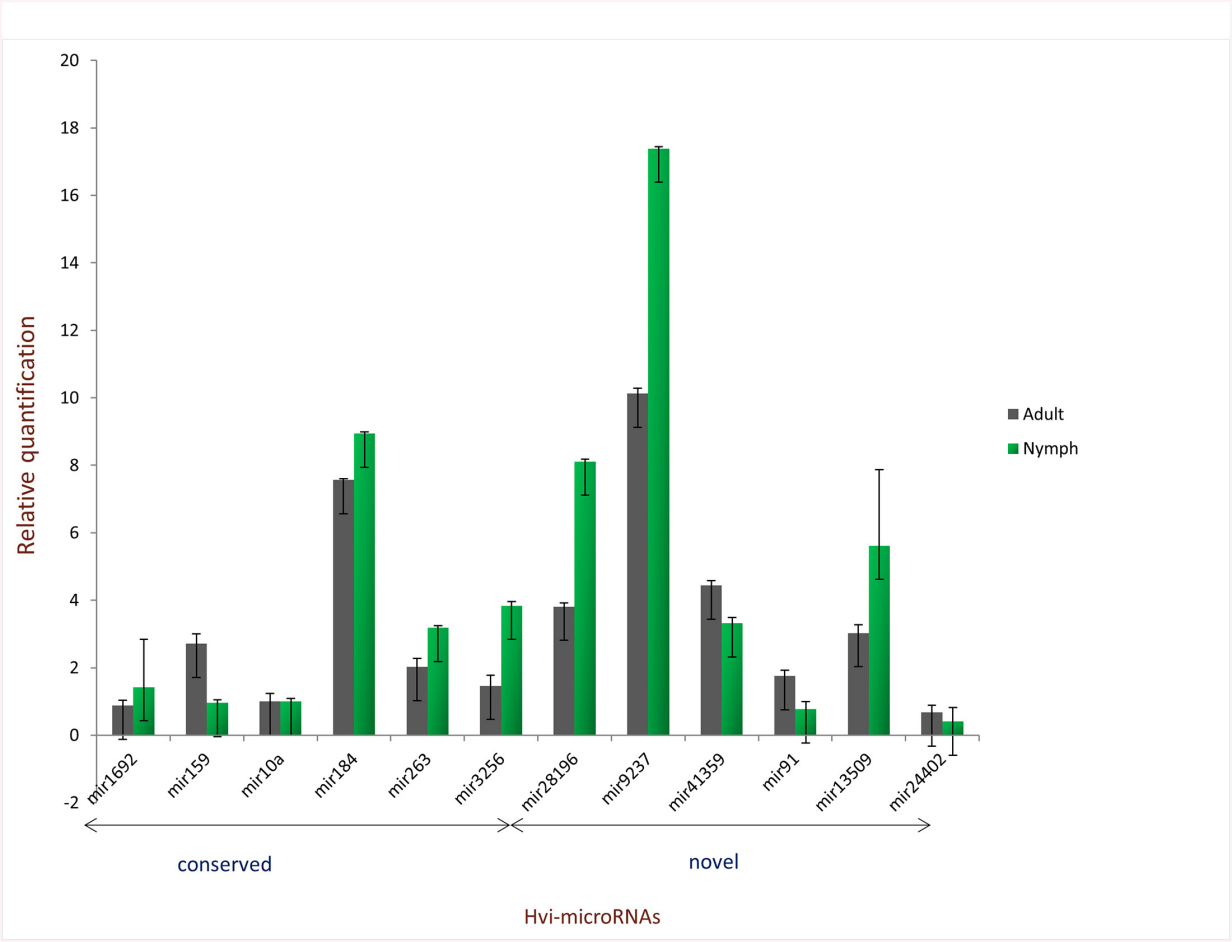
Quantitative stem-loop RT-PCR validation for conserved and novel microRNAs. The relative expression of the conserved and novel microRNAs of *H*. *vitripennis* were determined in adults and nymphs (2^nd^ and 3^rd^ instar) of *H*. *vitripennis*.

Based on our analysis, we were able to identify conserved and novel microRNAs from adult *H*. *vitripennis* (Fig 1 and Table 1). Further validation of their expression was done using stem-loop quantitative RT-PCR and microRNA northern blots. We tested six of the different microRNAs using the primer sets described in S1 Table and Fig 2. We were able to validate the expression of six of the conserved microRNAs from *H*. *vitripennis* (Hvi-miR1692, Hvi-miR159, Hvi-miR10a, Hvi-miR184, Hvi-miR263, Hvi-miR3256) and six novel microRNAs (Hvi-miR28196, Hvi-miR9237, Hvi-miR41359, Hvi-miR91, Hvi-miR13509 and Hvi-miR24402) through stem-loop quantitative RT-PCR (Fig 2). We were also able to detect the microRNAs in nymphal (2^nd^ and 3^rd^ instars) *H*. *vitripennis* (Fig 2).

**Fig 3:**
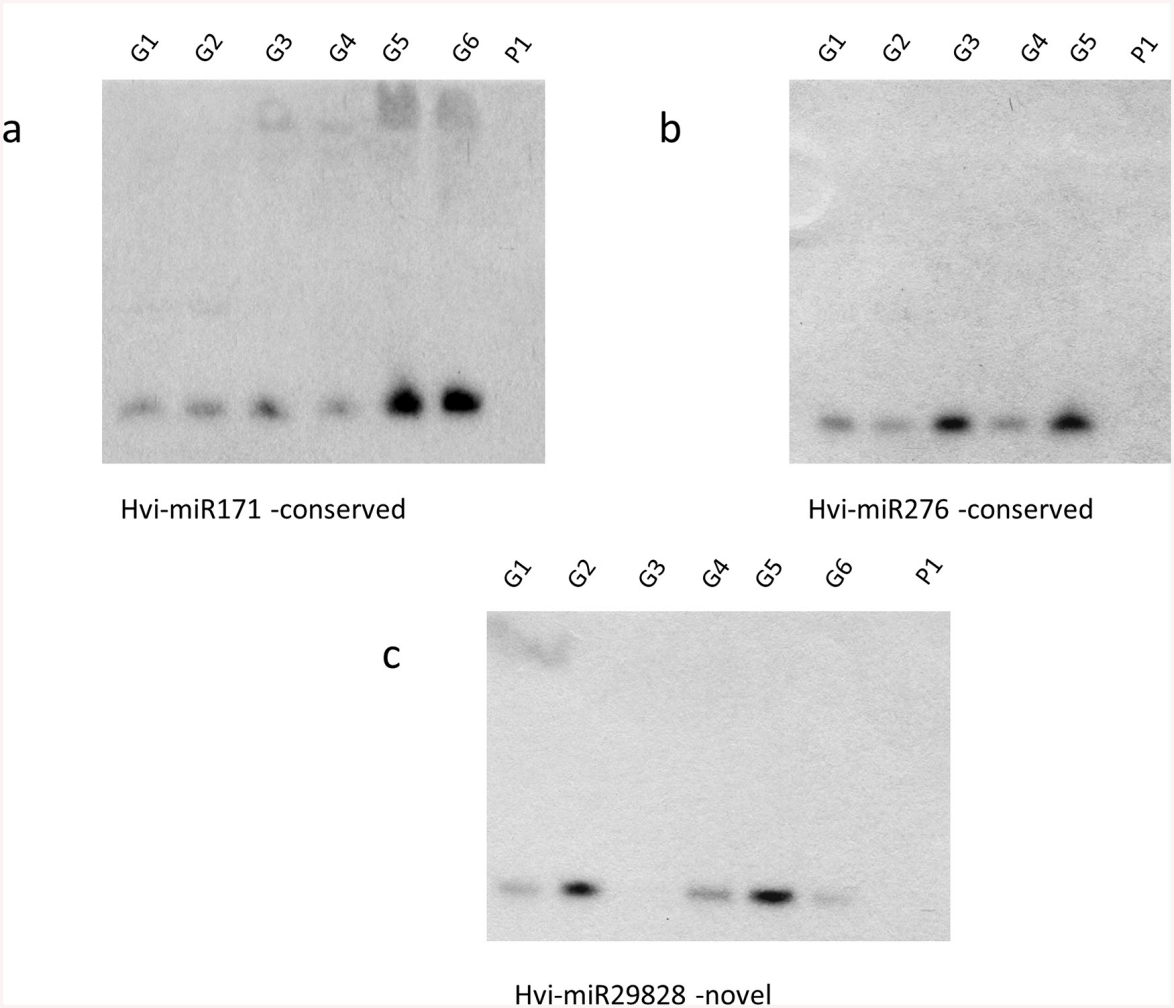
MicroRNA northern blot validation for conserved and novel microRNAs. Small RNAs (3 μg) were collected from individual adult *H*. *vitripennis* (lanes G1-G6) and separated on a 15% acrylamide–8 M urea gels and transferred to a nylon membranes. The small RNAs were then hybridized with 5’-end labeled oligomer probes that corresponded to the complementary sequences of Hvi-miR171 **(a)**, Hvi-miR276 **(b)**, and Hvi-miR29828 **(c)**. Hvi-miR171 and Hvi-miR276 are conserved microRNAs whereas Hvi-miR29828 is a novel microRNA identified in this study. Annotation: Lane P1, negative control potato small RNA sample.

The expression of Hvi-miR171, Hvi-miR276, and Hvi-miR29828 was validated by northern hybridization of small RNAs isolated from individual adult *H*. *vitripennis* (Fig 3). The expression of the conserved microRNAs (Hvi-miR171, Hvi-miR276, and Hvi-miR29828) was confirmed in all of the adults tested (5 or 6 individuals). The northern blot results validated the expression of these conserved and novel microRNAs in adult *H*. *vitripennis* (Fig 3).

### Transcriptome wide survey of the potential microRNA targets in *H*. *vitripennis*

MicroRNAs are known to regulate the mRNA levels thus influencing the development and immunity of the host organism [48–51]. The majority of microRNAs in animals are shown to interact with their targets through their 3’ and 5’-untranslated region (UTRs). In order to further understand the biological functions of novel microRNAs in adult *H*. *vitripennis*, we screened the transcriptome of *H*. *vitripennis* [15] for the potential miRNA targets (~52,700 putative mRNAs) using the microRNA target prediction program Miranda 3.3 with threshold parameters as described in materials and methods [38]. On average, 9,132 potential targets of the 14 novel microRNAs were identified (Table 6). There were on average of 608 targets per novel microRNA (Table 6). The number of potential targets varied in range from 172 (Hvi-miR66) to 1,583 (Hvi-miR13059) (Table 6). Each microRNA was predicted to target multiple mRNAs; the top mRNA targets for each of the novel microRNAs along with their description and scores are shown in Table 7. The predicted targets of the novel microRNAs were involved in a diverse range of functions that include developmental processes, reproduction, enzymatic process, and ion channels (Table 7 and S2 Table).

**Table 6:** Number of Potential MicroRNA Targets within the Transcriptome of *H*. *vitripennis*.

miRNA	Number of Targets
Hvi-miR29035	629
Hvi-miR13059	1,583
Hvi-miR28196	333
Hvi-miR9237	285
Hvi-miR19117	774
Hvi-miR24402	242
Hvi-miR66	172
Hvi-miR41359	234
Hvi-miR29828	586
Hvi-miR2062	1,191
Hvi-miR20768	653
Hvi-miR91	412
Hvi-miR24657	503
Hvi-miR6225	368

**Table 7:** Potential Targets of the Novel MicroRNAs from *H*. *vitripennis*.

miRNA	Probable Target	Target Description	Score	Energy
Hvi-miR29035	Locus_11150_Transcript_1/1_Confidence_1.000_Length_796	Dihydropyridine calcium channel	145	-18.64
	Locus_16382_Transcript_1/1_Confidence_1.000_Length_331	erg28-domain protein	145	-13.31
	Locus_23301_Transcript_1/1_Confidence_1.000_Length_1082	Zinc finger protein like	145	-12.59
	Locus_25188_Transcript_1/1_Confidence_1.000_Length_1044	Polymerase interacting protein–2	145	-10.76
	Locus_31683_Transcript_1/1_Confidence_1.000_Length_2171	Serine protease	145	-13.29
Hvi-miR13059	Locus_10604_Transcript_3/4_Confidence_0.500_Length_771	Kda midgut protein	145	-13.41
	Locus_10627_Transcript_1/1_Confidence_1.000_Length_1753	Pancreatic triacylglycerol lipase-like	145	-11.02
	Locus_11388_Transcript_1/1_Confidence_1.000_Length_2384	Defective proventriculus	145	-10.21
	Locus_12460_Transcript_1/1_Confidence_1.000_Length_744	Trehalose transporter like	145	-13.20
	Locus_12784_Transcript_1/1_Confidence_1.000_Length_2910	Semaphoring 2a	145	-11.61
Hvi-miR28196	Locus_7844_Transcript_1/1_Confidence_1.000_Length_1860	Sodium calcium exchanger 1 like	145	-12.75
	Locus_14067_Transcript_1/1_Confidence_1.000_Length_1330	Short chain dehydrogenase	146	-10.50
	Locus_45564_Transcript_1/1_Confidence_1.000_Length_511	Arylsulfatase b	147	-13.98
Hvi-miR9237	Locus_62333_Transcript_1/1_Confidence_1.000_Length_483	DNA mismatch repair protein like	145	-10.33
	Locus_67376_Transcript_1/1_Confidence_1.000_Length_206	Proton-coupled AA transporter like	145	-11.43
	Locus_11035_Transcript_1/1_Confidence_1.000_Length_839	Armadillo repeat protein like	146	-10.88
	Locus_15019_Transcript_1/1_Confidence_1.000_Length_906	Synaptic vesicle protein	146	-12.62
	Locus_2792_Transcript_4/4_Confidence_0.455_Length_748	Cubilin	146	-13.87
Hvi-miR19117	Locus_11772_Transcript_1/1_Confidence_1.000_Length_1668	Prestin-like	145	-12.97
	Locus_11826_Transcript_1/1_Confidence_1.000_Length_638	Mitochondrial inner membrane protein	145	-11.48
	Locus_12873_Transcript_1/1_Confidence_1.000_Length_1575	Probable histone acetyltransferase myst1	145	-14.78
	Locus_14018_Transcript_1/1_Confidence_1.000_Length_881	Probable signal peptidase complex subunit 2	145	-17.45
	Locus_17140_Transcript_1/1_Confidence_1.000_Length_1264	27 kDa haemolymph protein	145	-13.48
Hvi-miR24402	Locus_64765_Transcript_1/1_Confidence_1.000_Length_323	Zinc finger protein 43 like	146	-10.03
	Locus_7815_Transcript_1/1_Confidence_1.000_Length_1897	Serine protease	147	-10.45
	Locus_17779_Transcript_1/1_Confidence_1.000_Length_555	UDP-glycosyl transferase like	148	-11.47
	Locus_927_Transcript_1/1_Confidence_1.000_Length_535	Transketolase-like	148	-13.42
	Locus_13122_Transcript_1/1_Confidence_1.000_Length_2768	Chromodomain helicase DNA binding protein	150	-12.91
Hvi-miR66	Locus_41580_Transcript_1/1_Confidence_1.000_Length_1213	Phospholipase like	145	-11.02
	Locus_8279_Transcript_1/1_Confidence_1.000_Length_1372	Thread matrix protein partial	145	-11.40
	Locus_74835_Transcript_1/1_Confidence_1.000_Length_233	Sterol desaturase	146	-12.66
	Locus_1053_Transcript_1/2_Confidence_1.000_Length_320	Saposin-related protein	147	-13.26
	Locus_12889_Transcript_1/1_Confidence_1.000_Length_1634	Zinc finger protein noc like	147	-19.57
Hvi-miR41359	Locus_12803_Transcript_1/1_Confidence_1.000_Length_2167	Gamma-tubulin complex component 2	145	-14.43
	Locus_16061_Transcript_1/1_Confidence_1.000_Length_1080	Cyclin 1	145	-10.12
	Locus_19739_Transcript_1/1_Confidence_1.000_Length_3115	Transcription factor coe1	145	-11.47
	Locus_35858_Transcript_1/1_Confidence_1.000_Length_358	cdk-activating kinase assembly factor	145	-12.26
	Locus_36787_Transcript_1/1_Confidence_1.000_Length_516	Ubiquitin ligase	145	-15.18
Hvi-miR29828	Locus_12342_Transcript_1/1_Confidence_1.000_Length_1041	Alpha-tocopherol transfer protein	145	-11.06
	Locus_14644_Transcript_1/1_Confidence_1.000_Length_640	Glucose dehydrogenase	145	-16.71
	Locus_16653_Transcript_1/1_Confidence_1.000_Length_947	Phosphoinositide	145	-13.18
	Locus_19537_Transcript_1/1_Confidence_1.000_Length_1408	Glucosyl glucuronosyl transferases	145	-14.50
	Locus_21260_Transcript_1/1_Confidence_1.000_Length_1846	Cleavage stimulation factor subunit 1	145	-15.36
Hvi-miR2062	Locus_14702_Transcript_1/1_Confidence_1.000_Length_461	Mical-like protein 2	145	-10.85
	Locus_15570_Transcript_1/1_Confidence_1.000_Length_2290	e3 ubiquitin protein ligase rnf8	145	-12.56
	Locus_15716_Transcript_1/1_Confidence_1.000_Length_618	RNA-binding protein 8a like	145	-12.59
	Locus_17535_Transcript_1/1_Confidence_1.000_Length_1001	Projectin-like protein	145	-10.85
	Locus_2943_Transcript_1/1_Confidence_1.000_Length_2397	Transferrin	145	-11.96
Hvi-miR20768	Locus_10872_Transcript_1/1_Confidence_1.000_Length_2425	Tyrosine-protein kinase like	145	-10.42
	Locus_1171_Transcript_1/1_Confidence_1.000_Length_1797	Multidrug resistance protein 2	145	-12.86
	Locus_1268_Transcript_1/1_Confidence_1.000_Length_1165	ATP-citrate synthase	145	-11.59
	Locus_13954_Transcript_1/1_Confidence_1.000_Length_1773	Stress-activated MAPK IP	145	-12.81
	Locus_14648_Transcript_1/1_Confidence_1.000_Length_968	GTP-binding protein ypt7-like	145	-11.96
Hvi-miR91	Locus_30436_Transcript_1/1_Confidence_1.000_Length_2025	Inhibitor of growth protein 3	145	-12.31
	Locus_41696_Transcript_1/1_Confidence_1.000_Length_1730	Guanylate cyclase	145	-10.58
	Locus_7884_Transcript_1/1_Confidence_1.000_Length_763	Unc–44	145	-11.43
	Locus_9553_Transcript_1/1_Confidence_1.000_Length_671	Isoform f	145	-11.04
Hvi-miR24657	Locus_17020_Transcript_1/1_Confidence_1.000_Length_4328	Blastoderm specific protein 25d	145	-10.45
	Locus_19074_Transcript_1/1_Confidence_1.000_Length_286	Pax-interacting protein 1	145	-10.76
	Locus_27940_Transcript_1/1_Confidence_1.000_Length_444	Piggyback transposable element derived	145	-10.70
	Locus_3231_Transcript_1/1_Confidence_1.000_Length_2216	Glucose dehydrogenase	145	-13.41
	Locus_44981_Transcript_1/1_Confidence_1.000_Length_251	Tyrosine kinase	145	-14.13
Hvi-miR6225	Locus_10430_Transcript_1/2_Confidence_1.000_Length_526	Ets domain containing protein	145	-10.00
	Locus_13597_Transcript_1/1_Confidence_1.000_Length_1466	Non-specific lipid transfer protein	145	-13.65
	Locus_1889_Transcript_1/5_Confidence_0.643_Length_603	Cytochrome c subunit	145	-10.14
	Locus_19266_Transcript_1/1_Confidence_1.000_Length_1728	Riboflavin transporter 2 like	145	-10.37
	Locus_21980_Transcript_1/1_Confidence_1.000_Length_1466	Fast kinase domain 1	145	-12.72

## Discussion

MicroRNAs form an important class of small RNAs that play important roles in insect development [8]. Here we identified 14 novel microRNAs from adult *H*. *vitripennis*. The precursors of these novel microRNAs were identified using previously determined and publicly available transcriptome information for *H*. *vitripennis*. These precursors were found to form low-energy, fold-back structures. The concise coordinates of the identified precursor microRNAs helped us to excise the precursor sequences for structural analysis. Furthermore, we also identified conserved microRNAs from *H*. *vitripennis* by homology analysis with microRNAs from other insects in miRBase (Fig 1).

Sequence analysis of the 14 novel microRNAs from *H*. *vitripennis* suggests a strong bias for a 5’ terminal uridine. This 5’ terminal bias was found in 11 of the 14 novel microRNAs that we identified (Hvi-miR29035, Hvi-miR13059, Hvi-miR28196, Hvi-miR9237, Hvi-miR19117, Hvi-miR24402, Hvi-miR66, Hvi-miR41359, Hvi-miR29828, Hvi-miR20768 and Hvi-miR91). In two other microRNAs (Hvi-miR2062 and Hvi-miR6225) we observed a guanosine bias whereas Hvi-miR24657 showed a bias for adenosine. This difference in the 5' terminal nucleotide leads to a loading bias of the microRNA into different Argonaute proteins: a 5’ uridine containing microRNA loads into AGO1, while one with a 5’ adenosine loads into AGO2, and one with cytidine at the 5’ terminus loads into AGO5 [6]. Furthermore, the strong 5’ bias for uridine also suggests selective loading of the mature strand microRNA over the star strand. This is consistent with the hypothesis that only one of the strands enters into the RISC complex [6].

Our datasets were generated from total RNA that was isolated from the whole body of adult *H*. *vitripennis* and hence may lack the specificity, depth, and resolution that is needed for more robust analysis of temporal and spatial expression of the microRNAs. Since the focus of our study was the adult stage of *H*. *vitripennis*, the expression pattern of the microRNAs in the juvenile and embryonic stages was not determined. Conserved microRNAs are dominant in late stage embryos of *D*. *melanogaster* and *D*. *virilis*, whereas in the early embryonic stages rapidly evolving microRNAs are more common [52]. Thus, both the conservation (i.e., whether conserved or novel) and type (i.e., whether mature or star strand) of microRNA that is present at a particular time can differ depending upon the developmental stage of the organism. Our selection of only a single developmental stage of *H*. *vitripennis* may partly explain the generally low occurrence of star strand reads in our library (Table 5). Recent studies also suggest that the presence of star strands is more common in eukaryotes and vertebrate organisms [5] and that these star strands may function as mature microRNAs [53]. Consistent with these studies is the report of the enhanced abundance of certain star strands in microRNA libraries from *Manduca sexta* [30] and the zebra finch [53]. It will be interesting to characterize the novel microRNAs of *H*. *vitripennis* with respect to the developmental stage and under different physiological conditions.

Interestingly, the putative origin of 7 out of the 14 identified novel microRNAs (Hvi-miR13059, Hvi-miR9237, Hvi-miR19117, Hvi-miR24402, Hvi-miR41359, Hvi-miR91 and Hvi-miR24657) was found within a transposable element sequences. Insect genomes are substantially loaded with transposable elements, and their presence helps to explain the varying sizes of their genomes. In particular, insect genomes carry widely varying amounts of repeat sequences in their genomes [54]. In *H*. *vitripennis*, we previously identified four different types of transposable elements with a majority of them belonging to TC3 type (131 copies) and *piggyBac* elements (58 copies) [15]. Recent evidence suggests that the functional evolution of microRNAs is partly due to insertions by the transposable elements resulting in the generation of novel microRNAs [55–57].

MicroRNAs influence the development and immunity of the host organism by regulating mRNA levels [48–51]. The majority of microRNAs in animals are shown to interact with their target mRNAs through the 3’- and 5’-untranslated regions (UTRs) of the mRNAs. MicroRNAs are thus believed to regulate their target genes at a post-transcriptional level. We used a library of full-length transcripts (~52,700 putative mRNAs) of *H*. *vitripennis* for *in silico* target prediction. We identified 9,132 potential microRNA targets through the Miranda target prediction program (Tables 6 and 7, S2 and S3 Tables). Several key regulatory genes, as well as genes involved with signaling pathways, ion channels, enzymatic processes, catabolic reactions and KEGG pathways were identified by this process. The future validation and further study of these potential targets will lead to a deeper understanding the regulatory biology of *H*. *vitripennis* and likely lead to the identification of novel gene targets that can be exploited for control of this pest insect.

## Conclusions

In the present study, we identified 14 novel microRNA candidates from *H*. *vitripennis*. To date, this is the first report on such an analyses in *H*. *vitripennis*. These data provide a promising approach in gene functional studies through the use of RNAi-based approaches in cell lines or whole insects. The expression of microRNAs that target key regulatory genes in *H*. *vitripennis* through transgenic plants has potential to control *H*. *vitripennis* and/or the pathogens that it vectors.

## Supporting Information

S1 Table Primers used for the amplification of microRNAs from *H*. *vitripennis* by real time PCR. (DOCX)

S2 Table Predicted microRNA targets from *H*. *vitripennis* whole adults through the use of the Miranda target prediction program. (CSV)

S3 Table Raw output of the predicted microRNA targets from *H*. *vitripennis* through the use of the Miranda target prediction program. (ZIP)
