# Supplementary material for: Identification of Novel and Conserved microRNAs in Homalodisca vitripennis, the Glassy-Winged Sharpshooter by Expression Profiling
Source: PLoS One. 2015 Oct 6;10(10):e0139771. doi: 10.1371/journal.pone.0139771 (PMC4595010; doi:10.1371/journal.pone.0139771)
Supplement: S1 Table — (DOCX) [file pone.0139771.s001.docx]

**Table S1**. Primers designed for the amplification of micro RNAs from *Homalodisca vitripennis* whole adults through the use of real time PCR

| **Primer Name** | **Sequence (5'-3')** |
| --- | --- |
| Universal reverse | GTGCAGGGTCCGAGGT |
| StemLoop RT | GTCGTATCCAGTGCAGGGTCCGAGGTATTCGCACTGGATACGA*NNNNNN* |
| miR171 for | GCGGCGGTGATTGAGCCGCGCC |
| miR159 for | GCGGCGGTTTGGATTGAAGGGA |
| miR281 for | GCGGCGGTGTCATGGAATTGC |
| miR276 for | GCGGCGGTAGGAACTTCATAC |
| miR10a for | GCGGCGGTACCCTGTAGATCC |
| miR184 for | GCGGCGGTGGACGGAGAAC |
| miR1692 for | GCGGCGGTGTAGCTCAGTTG |
| miR71 for | GCGGCGGTGAAAGACATGGG |
| miR3256 for | GCGGCGGTGGCTCTGATTTCC |
| miR263a for | GCGGCGGAATGGCACTGAAAG |
| miR9237 for | GCGGCGGTGTTTGAACACCTC |
| miR28196 for | GCGGCGGTAGTTTACATCATC |
| miR29828 for | GCGGCGGTCTGCTTGCTCCG |
| miR13509 for | GCGGCGGTTTGTTCTGAATGG |
| miR24402 for | GCGGCGGTTATAGTTCCTTGC |
| miR41359 for | GCGGCGGTAGATCTAGTTCC |
| Ubiquitin for | CAAGACCCTGACTGGCAAGAC |
| Ubiquitin rev | GCCTCCACGAAGACGGAGAAC |

The sequences italicized and underlined were specific for each of the micro RNA and represents the last six nucleotides of the sequence.
